# Editorial: Highlights in elite sports and performance enhancement 2021/22

**DOI:** 10.3389/fspor.2023.1150417

**Published:** 2023-03-02

**Authors:** Kazushige Goto, Gustavo R. Mota, Stéphane Bermon

**Affiliations:** ^1^ Faculty of Sport and Health Science, Ritsumeikan University, Kusatsu, Japan; ^2^ Exercise Science, Health and Human Performance Research Group, Department of Sport Sciences, Institute of Health Sciences, Federal University of Triângulo Mineiro, Uberaba, Brazil; ^3^ World Athletics, Health and Science Department Monaco, Monaco, Monaco; ^4^ Laboratoire Motricité Humaine Expertise Sport Santé, Université Côte d’Azur, Nice, France

**Keywords:** exercise, physical training, olympics, team-sports, recovery

**Editorial on the Research Topic** Highlights in elite sports and performance enhancement 2021/22

## 1. Introduction

Elite sports and performance enhancement are deeply associated. Sports scientists and coaches are always observing, evaluating, planning, and acting to improve their results. In this sense, the evidence-based practice approach is relevant. Therefore, the current research topic (RT) – Highlights in Elite Sports and Performance Enhancement 2021/22 has the aim to deliver a selection of high-impact articles written by leaders in the field. A diversity of research across the Elite Sports and Performance Enhancement section is spotlighted.

## 2. Articles

The current RT has nine articles in total. Five original research articles (Poignard et al.; Dimundo et al.; Höög and Andersson; James et al.; Riazati et al.), one opinion article (Brocherie and Beard), one systematic review (Millet et al.), one brief research report (Kubayi and Larkin), and one perspective article (Almqvist et al.).

Poignard et al. studied the impact of recovery practices in tennis players and found that a combination of 2–3 techniques has been used. However, cooling techniques were the most widely used modality and attenuated muscle soreness, regardless of the training type (Poignard et al.).

James et al. reported the volume and intensity of locomotor activities in international men's field hockey matches over a 2-year period. The first playing quarter had the highest physical demands, but match physical demands varied by playing position. Also, there was a strong negative relationship between total playing time and the intensity of locomotor activities. These data may help to inform training, recovery, and planned substitutions practices (James et al.).

Höög and Andersson described the physical capacity of elite TeamGym athletes and analyzed difference between sex and experience level (i.e., junior vs. senior athletes). Their results showed males performed better than females and seniors performed better than junior TeamGym athletes (Höög and Andersson).

Through a multidisciplinary research approach, Dimundo et al. investigated the attributes involving selection in under-15 rugby union players at an English Premiership Regional Academy. The authors concluded that anthropometric and physiological qualities (e.g., body mass, strength, and speed) are better explained selection than cognitive aspects and birth quartiles (Dimundo et al.).

Riazati et al. investigated the time course of recovery for gait and neuromuscular function immediately after and 24-h post-interval training. They found that following a high intensity interval training session, master runners experienced significant impairments to both central and peripheral drive, besides changes in gait kinematics and force. Although most of the runners recovered within 24-h, a small number did not. These authors concluded that waiting for a full recovery after running may help to prevent overuse injuries (Riazati et al.).

To bridge the gap between research and practice in sports, Brocherie and Beard suggested a global approach. Among other things, they suggested improving collaboration with coaches/managers and athletes, establishing trust and building relationships with academics or other sports industry infrastructures, and improving quality decisions in practice (Brocherie and Beard).

Millet et al. performed an extensive and complex bibliometric analysis of research involving all summer and winter Olympic Sports. Interestingly, nine (i.e., 18%) out of 50 Olympic sports represented 17,252 articles out of 25,003 articles in total, corresponding to 69% of all selected publications (Millet et al.).

Kubayi and Larkin studied the 2019 Africa Cup of Nations Soccer Championship focusing on the match statistics associated with success. Their findings showed that the winning teams presented a greater number of total shots, shots on target, and shots from counter-attacks compared to the losing squads (Kubayi and Larkin).

Almqvist et al. presented a scientific perspective on reducing ski-snow friction to improve performance in Olympic cross-country skiing. The authors highlight that performance on “narrow skis” has improved extensively in recent decades and that future insights into how best to reduced ski-snow friction could allow for additional advancements (Almqvist et al.).

## 3. Final considerations

The current RT highlights the importance of evidence-based practices in improving sports performance. The articles included in this RT reveal the variety of research in the field. The studies range from investigations into recovery practices, physical capacities, attributes, the time course of recovery, and performance improvements. The findings provide new insights and contribute to a better understanding of sports performance enhancement. The importance of improving collaboration between researchers, coaches, athletes, and sports industry infrastructures was also emphasized. This RT offers useful information for sports scientists and coaches, as they strive to improve sports performance.

